# Primary health care nursing interventions for people who use drugs: a scoping review

**DOI:** 10.15649/cuidarte.5254

**Published:** 2026-10-14

**Authors:** Lina Marcela Acuña-Forero, Yina Lorena Sosa-Rodríguez, Fernando José Cárdenas-Martínez, Javier Mauricio Sánchez-Rodríguez

**Affiliations:** 1 Fundación Universitaria Sanitas, Bogotá, Colombia. E-mail: lm.acunafo@unisanitas.edu.co Fundación Universitaria Sanitas Bogotá Colombia lm.acunafo@unisanitas.edu.co; 2 Fundación Universitaria Sanitas, Bogotá, Colombia. E-mail: yl.sosaro@unisanitas.edu.co Fundación Universitaria Sanitas Bogotá Colombia yl.sosaro@unisanitas.edu.co; 3 Facultad de Enfermería, Fundación Universitaria Sanitas, Bogotá, Colombia. E-mail: fernando.cardenas@metropol.gov.co Fundación Universitaria Sanitas Bogotá Colombia fernando.cardenas@metropol.gov.co; 4 Docente Facultad de Ciencias de la Salud, Universidad Autónoma de Bucaramanga, Bucaramanga; Facultad de Enfermería, Fundación Universitaria Sanitas, Bogotá, Colombia. E-mail: jsanchez477@unab.edu.co Fundación Universitaria Sanitas Bogotá Colombia jsanchez477@unab.edu.co

**Keywords:** Nursing, Primary Health Care, Harm Reduction, Psychotropic Drugs, Enfermería, Atención Primaria en Salud, Reducción de Daños, Psicotrópicos, Enfermagem, Atenção Primária à Saúde, Redução do Dano, Psicotrópicos

## Abstract

**Introduction:**

Drug use is a public health concern due to its high prevalence and associated biopsychosocial consequences. Despite theimportanceofharmreductioninprimaryhealthcare, aknowledge gap exists regarding the specific interventions implemented by nurses.

**Objective:**

To describe the nursing interventions that can be implemented within primary health care for people who use drugs to reduce harm associated with drug use.

**Materials and Methods:**

A scoping review was conducted following the Arksey and O’Malley framework. The literature search was conducted in PubMed, VHL, ProQuest, and Google Scholar in July 2024.

**Results:**

A total of 1,518 studies were identified; of which 12 met the eligibility criteria for analysis: 10 retrieved from databases and 2 from Google Scholar. Five categories emerged from the analysis, of which educational/ psychoeducational interventions were the most frequently reported (75%), followed by preventive and risk-mitigation practices (50%) and clinical and emotional support interventions (42%).

**Discussion:**

The role of nursing in reducing harm from drug use is highlighted, given that interventions ranging from education and psychological support through cognitive-behavioral therapy to preventive actions and clinical risk mitigation practices contribute to the well-being and care of each person.

**Conclusion:**

Nursing interventions in primary health care are key to improving quality of life and require specific training for professionals and policies that facilitate access to harm reduction resources.

## Resumen

**Introducción:**

El consumo de drogas es un evento de interés en salud pública por su alta incidencia y las consecuencias biopsicosociales asociadas. A pesar de la relevancia de la reducción de daños en atención primaria, existe una brecha de conocimiento con respecto a las intervenciones específicas de enfermería.

**Objetivo:**

Describir las intervenciones de Enfermería que se pueden realizar desde la Atención Primaria en Salud en personas consumidoras de drogas para la reducción de los daños asociados al consumo.

**Materiales y métodos:**

Revisión de alcance siguiendo el marco de Arksey y O'Malley, se realizó la búsqueda en PubMed, BVS, ProQuest y Google Scholar, en julio de 2024.

**Resultados:**

Se identificaron 1.518 estudios, de los cuales 12 cumplieron criterios para su análisis: 10 provenientes de bases de datos y 2 de Google Scholar. Emergieron cinco categorías, de las cuales las intervenciones educativas/psicoeducativas fueron las más reportadas con 75%, seguido de las preventivas y prácticas orientadas a la mitigación de riesgos con 50%, y las intervenciones clínicas y las de apoyo emocional con 42%.

**Discusión:**

Se destaca el rol de Enfermería en la reducción de daños por consumo de drogas dado que las intervenciones que van desde la educación, el apoyo psicológico mediante terapia cognitivo-conductual, hasta acciones de prevención y prácticas clínicas de mitigación de riesgos, contribuyen con el bienestar y cuidado de cada persona.

**Conclusiones:**

Las intervenciones de Enfermería en Atención Primaria en Salud son clave para mejorar la calidad de vida, requiriendo capacitación específica de los profesionales y políticas que faciliten el acceso a recursos para la reducción de daños.

## Resumo

**Introdução:**

O uso de drogas é uma preocupação de saúde pública devido à sua alta incidência e às consequências biopsicossociais associadas. Apesar da importância da redução de danos na atenção primária, existe uma lacuna de conhecimento em relação a intervenções específicas de enfermagem.

**Objetivo:**

Descrever as intervenções de enfermagem que podem ser implementadas na atenção primária à saúde para usuários de drogas, visando reduzir os danos associados ao seu uso.

**Materiais e métodos:**

Foi realizada uma revisão de escopo seguindo a estrutura de Arksey e O'Malley. As buscas foram realizadas no PubMed, BVS, ProQuest e Google Scholar em julho de 2024.

**Resultados:**

Foram identificados 1.518 estudos, dos quais 12 atenderam aos critérios de inclusão para análise: 10 de bases de dados e 2 do Google Scholar. Cinco categorias emergiram, sendo as intervenções educativas/ psicoeducacionais as mais frequentemente relatadas (75%), seguidas por práticas preventivas e de mitigação de riscos (50%) e intervenções de apoio clínico e emocional (42%).

**Discussão:**

Destaca-se o papel da enfermagem na redução de danos relacionados ao uso de drogas, visto que intervenções que vão desde a educação e o apoio psicológico, passando pela terapia cognitivo-comportamental, até práticas clínicas de prevenção e mitigação de riscos, contribuem para o bem-estar e o cuidado de cada indivíduo.

**Conclusões:**

As intervenções de enfermagem na atenção primária à saúde são fundamentais para a melhoria da qualidade de vida, exigindo formação específica para os profissionais e políticas que facilitem o acesso a recursos de redução de danos.

## Introduction

Drug use is a phenomenon of growing concern worldwide. According to the Global Drug Report 2023, approximately 296 million users were recorded in 2021, representing a 23% increase over the past decade. Among the most commonly used drugs, cannabis tops the list with 219 million users, followed by amphetamines (36 million) and cocaine (22 million) worldwide^1^.

In Latin America and the Caribbean, at least 4.4 million men and 1.2 million women have experienced drug-related disorders at some point in their lives^2^,^3^. In Colombia, alcohol and tobacco remain the most prevalent substances, with consumption rates of 88.84% and 50.39%, respectively. However, there has been an increase in the use of opioids without a prescription, rising from 1.38% in 2016 to 3.94% in 2022^2^,^3^.

The impact of drug use extends beyond the individual, affecting public health, safety, and social cohesion. Psychological, social, and environmental factors influence the onset and continuation of drug use, highlighting the need to address this issue through a comprehensive approach^4^, given that health consequences range from cardiovascular diseases, neuropsychological disorders, and cognitive decline to an increased risk of communicable infections such as human immunodeficiency virus (HIV) and hepatitis C^5^,^6^.

Despite the increased demand for care for people with problematic drug use, training for health personnel, especially in the nursing field, remains limited, which hinders the implementation of effective prevention and harm reduction strategies^7^.

Furthermore, harm reduction has proven to be a strategy based on systematic, evidence-based interventions to minimize the consequences associated with drug use by promoting access to health services, reducing morbidity and mortality, and mitigating the social impact of this problem^8^. Programs such as naloxone distribution^9^,^10^, supervised consumption sites, and substitution treatments have shown positive results in reducing overdoses and preventing diseases^11^,^12^.

In Colombia and Latin America, drug use is on the rise, and coverage of these programs remains insufficient, highlighting the need to strengthen public policies and the training of health personnel for the implementation of strategies and interventions in this area^13^,^14^.

In this context, nursing plays a crucial role in the care of people with problematic drug use. Within Primary Health Care (PHC), nursing professionals can play a key role in the prevention, early detection, and timely follow-up of these cases^15^,^16^. Training in harm reduction strategies not only improves the quality of care but also contributes to destigmatization and equitable access to health services^17^,^18^. Additionally, there is a lack of consistency in the literature on specific interventions for this discipline in this context; therefore, a scoping review would allow for mapping and synthesizing available knowledge, and identifying research gaps. This aims to achieve a better approach to health from a more humanistic and inclusive perspective within health systems.

Therefore, the objective of this review is to describe nursing interventions that can be implemented within primary health care (PHC) for people who use drugs to reduce harm associated with drug use, as reported in the scientific literature.

## Materials and Methods

This article is a scoping review, which was conducted following the methodological framework proposed by Arksey and O’Malley and adapted by Levac, Colquhoun, and O’Brien^19^.

**Identification of the Research Question**

Initially, the research question “What interventions do primary health care nursing professionals implement to reduce harm associated with drug use?” was identified using the PIO framework.

**Identification of Relevant Studies**

To identify the studies, a search was conducted in the following databases—PubMed/Medline, the Virtual Health Library (VHL), and ProQuest—in July 2024, using a specific search strategy. The search strategy was carried out using Boolean operators (AND, OR, NOT) and Medical Subject Headings (MeSH) and Descriptores en Ciencias de la Salud (DeCS) terms available in the Virtual Health Library (VHL). As an additional source, a sensitive search strategy was conducted in Google Scholar using the terms: ("Drug Users" + "primary care nursing" + "harm reduction"). The search strategies used in each database are presented in Table 1.

**Table 1:** Final search strategy specific to each database

**PUBMED**
(((((((((Substance Abuse, Oral[Title/Abstract]) OR (Substance Abuse, Oral[MeSH Terms])) OR (Drug Users[Title/Abstract])) OR (Drug Users[MeSH Terms])) OR (Substance-Related Disorders[Title/Abstract])) OR (Substance-Related Disorders[MeSH Terms])) OR (Psy-chotropic Drugs[Title/Abstract])) OR (Psychotropic Drugs[MeSH Terms])) AND ((((((((((Nursing[Title/Abstract]) OR (Nursing[MeSH Terms])) OR (Nursing Care[Title/Abstract])) OR (Nursing Care[MeSH Terms])) OR (Nurses[Title/Abstract])) OR (Nurses[MeSH Ter-ms])) OR (primary care nursing[Title/Abstract])) OR (primary care nursing[MeSH Terms])) OR (Primary Nursing[Title/Abstract])) OR (Primary Nursing[MeSH Terms]))) AND ((((((((((harm reduction[Title/Abstract]) OR (harm reduction[MeSH Terms])) OR (Risk Re-duction Behavior[Title/Abstract])) OR (Risk Reduction Behavior[MeSH Terms])) OR (Disaster Management[Title/Abstract])) OR (Di-saster Management[MeSH Terms])) OR (Risk Management[Title/Abstract])) OR (Risk Management[MeSH Terms])) OR (Risk Evalua-tion Mitigation[Title/Abstract])) OR (Risk Evaluation and Mitigation[MeSH Terms]))
**Virtual Health Library**
((nursing) OR (mh:(nursing)) OR (nursing care) OR (mh:(nursing care)) OR (nurses) OR (mh:(nurses)) OR (primary care nursing) OR (mh:(primary care nursing)) OR (primary nursing) OR (mh:(primary nursing))) AND ((substance abuse, oral) OR (mh:(substance abuse, oral)) OR (substance abuse, oral) OR (mh:(substance abuse, oral)) OR (drug users) OR (mh:(drug users)) OR (substance-related disorders) OR (mh:(substance-related disorders)) OR (psychotropic drugs) OR (mh:(psychotropic drugs))) AND ((harm reduction) OR (mh:(harm reduction)) OR (risk reduction behavior) OR (mh:(risk reduction behavior)) OR (disaster management) OR (mh:(disaster management)) OR (risk management) OR (mh:(risk management)) OR (risk evaluation AND mitigation) OR (mh:(risk evaluation AND mitigation)))
**ProQuest**
(noft(Substance Abuse, Oral) OR subject(Substance Abuse, Oral) OR noft(drug users) OR subject(Drug Users) OR noft(Substance-Re-lated Disorders) OR subject(Substance-Related Disorders) OR noft(Psychotropic Drugs) OR subject(Psychotropic Drugs)) AND (noft(-Nursing) OR subject(Nursing) OR noft(Nursing Care) OR subject(Nursing Care) OR noft(Nurses) OR subject(Nurses) OR noft(primary care nursing) OR subject(primary care nursing) OR noft(Primary Nursing) OR subject(Primary Nursing)) AND (noft(harm reduction) OR subject(harm reduction) OR noft(Risk Reduction Behavior) OR subject(Risk Reduction Behavior) OR noft(Disaster Management) OR subject(Disaster Management) OR noft(Risk Management) OR subject(Risk Management) OR noft(risk evaluation AND mitigation) OR subject(risk evaluation AND mitigation))

**Study Selection**

During the literature review, the following inclusion criteria were considered: articles involving nursing interventions; articles involving people who use drugs as the target population; articles addressing primary health care (PHC); articles focused on harm reduction; articles published in Spanish, English, Portuguese, and French; and articles with full-text availability. The exclusion criteria were theoretical review articles, opinion pieces, commentaries, editorials, letters, conference abstracts, case reports or case series, and articles in which the study population had an additional condition besides drug use (e.g., chronic noncommunicable diseases, cardiovascular diseases, depression, among others).

Furthermore, no time restrictions were applied to the database searches to avoid publication and information bias.

Subsequently, the search results were exported from each database, and study selection was performed using the Rayyan QCRI^20^. In the first stage, duplicate records were removed independently by two reviewers. Next, each article underwent title and abstract screening to identify discrepancies in the inclusion and exclusion criteria, which served as the basis for information screening. These discrepancies were resolved jointly by consensus between the two reviewers. The full-texts of the selected articles were then retrieved for subsequent verification and analysis. The selection process was reported using the PRISMA flowchart.

**Data Charting**

To extract data from each study, a Microsoft Excel® data charting was developed to collect and organize the data, with the aim of extracting the following variables: title, author, year of publication, country where the study was conducted, study design, study objective, population, and type of intervention.

**Collating, summarizing, and reporting the results**

For reporting the results, the PRISMA Extension for Scoping Reviews (PRISMA-ScR) recommendations were followed^21^. First, the study selection phase is presented, followed by the individual characteristics of the included articles through a summary figure. Some articles were found to share the risk reduction approach, leading to the creation of 5 categories to group the interventions; however, each intervention was also analyzed individually. The information extracted from the studies and supplementary material is available on the Figshare platform^22^.

**Ethical Considerations**

This study is classified as research without ethical risk, since all sources used have been properly cited in accordance with the principles of academic rigor and the ethical principles set forth in Resolution 8430 of 1993^23^. Therefore, it did not require approval from the Ethics Committee, in accordance with local regulations.

## Results

**Identification of Studies**

A total of 1,518 articles were initially identified. After duplicate removal and title and abstract screening, 57 articles remained for full-text review, of which 47 were excluded, leaving 10 studies for analysis. In addition, 77 studies were identified in other sources (Google Scholar), of which only 2 met the established criteria, resulting in a total of 12 articles included in the review^24^–^35^. Details are presented in Figure 1.

**Figure 1:**
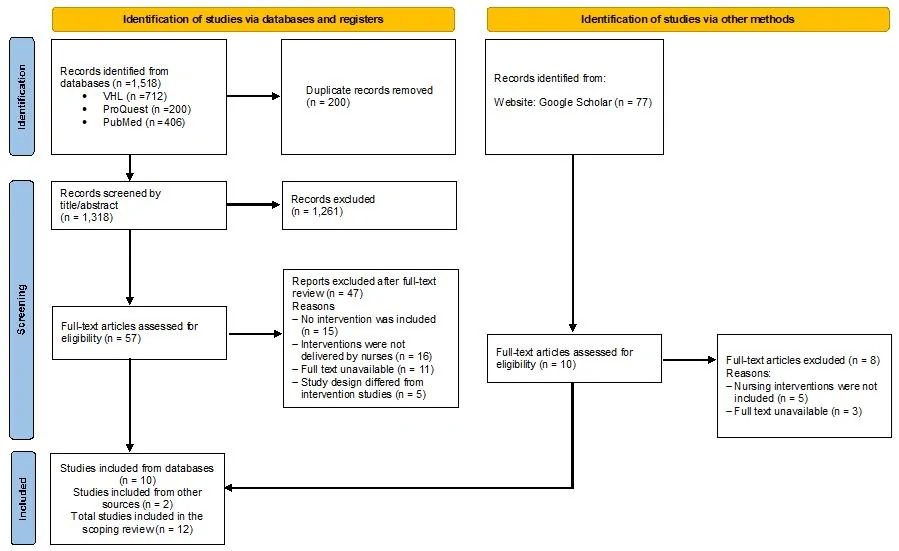
PRISMA flow diagram of the article search and selection process

**Study characteristics**

The identified studies were conducted primarily in the United States, in states such as New Jersey and California (6), and to a lesser extent from Asia (1), South Africa (1), Toronto/Canada (1), Australia (1), Spain (1), and Cuernavaca/Mexico (1). The publications spanned the period from 2001 to 2022 and employed various methodological approaches: quantitative (8/12), mixed-methods (3/12), and qualitative (1/12).

**Individual characteristics of the studies**

The individual characteristics of the articles highlight the importance of the role of nursing professionals in the care of drug-using patients in clinical settings, educational settings, and supervised consumption sites; these results are shown in Table 2.

**Characteristics of interventions for people who use drugs**

In the field of healthcare, strategies for managing drug use and harm reduction are essential to mitigate its negative effects. Through clinical and community-based interventions, healthcare professionals implement various actions aimed at preventing overdoses, reducing the transmission of infectious diseases, offering timely treatments, and providing comprehensive support to people with addiction problems.

Therefore, based on the interventions identified in the reviewed articles, five main categories were defined, which are summarized in Figure 2 and described in detail in Supplementary Material 1 .

**Table 2:** Individual characteristics of the studies

Author/year/ country	Study objective	Study design	Population	Type of intervention
Cataldo^31^ 2001 USA	To highlight the importance of the role of psychiatric nurses in the assessment and treatment of tobacco use.	Quantitative descriptive	Psychiatric patients and individuals with substance use disorders	• Pharmacological treatment to quit smoking. • Individualized counseling to quit smoking. • Education on the risks and benefits of quitting smoking. • Ongoing follow-up and emotional support for patients.
WHO^25^ 2008 Asia	Describe the management of health problems in drug users	Descriptive	Healthcare professionals	• Medical treatment for infections and diseases common among drug users. Vaccination against hepatitis and tetanus. • Provision of clean needles and sterile injection equipment. • Education on the prevention of infectious diseases.
Pengpid et al.^26^, 2013 South Africa	To evaluate the effectiveness of a Screening and Brief Intervention- SBI for alcohol problems among university students in South Africa.	Quantitative study - controlled trial	College students	• Screening to identify problematic alcohol use. • Brief intervention to discuss strategies for reducing excessive alcohol consumption. • Information-Motivation-Behavioral Skills (IMB) model.
Burr et al.^30^, 2014 USA / New Jersey	To describe the implementation of a nurse-led health promotion and disease prevention program in needle exchange programs.	Quantitative Descriptive	People who inject drugs	• Provision of clean syringes and safe injection equipment. • Access to medical care and HIV/hepatitis testing. • Education on safe injection practices and overdose prevention.
Patestos et al.^27^, 2014 USA	Analyzes the roles of school nurses in preventing substance abuse among school-aged children through a model of continuous care.	Quantitative descriptive	School-aged children and adolescents	• Early detection of substance abuse problems. • Educational programs in schools on the risks of drug use. • Connecting students with counseling or treatment services.
Gutierrez et al.^24^, 2015 USA	To identify the current prevalence and societal impact, as well as the etiology, comorbid psychiatric disorders, and treatment and prevention of drug and alcohol use among adolescents.	Descriptive study	Adolescents	• Education on the harmful effects of alcohol and drug use. • Behavioral and family therapies. • Prevention of initiation of use through informational talks.
Sharma et al.^32^, 2017 Toronto Canada	Involving people with firsthand experience with drug use as partners in the delivery of clinical care.	Quantitative study	Adults and adolescents	• Provision of sterile supplies (syringes) to drug users. • Treatment of drug-related infections.
Lintzeris et al.^28^, 2020 Australia	Implement and evaluate a NHT intervention targeting individuals at high risk of opioid overdose, specifically those with a history of opioid use disorder and/or injection drug use.	Mixed-methods study	Adults and adolescents	• Distribution of naloxone and training on its use. • Education on how to identify signs of overdose.
Mullennix et al.^34^, 2020 USA	Describe the implementation of a clinical nurse specialist-led emergency department overdose education and naloxone distribution program.	Quantitative study	Patients of all ages who have experienced an opioid overdose or are at risk of opioid overdose	• Naloxone distribution in the emergency department. • Training for patients and family members on naloxone administration. • Screening of patients at risk of opioid overdose. • Education on the risks of overdose and opioid use. • Referral to opioid dependence treatment services. • Post-discharge follow-up to ensure access to care and treatment resources.
Clua-García^35^, 2021 Spain	Understanding nursing care in supervised consumption sites (SCSs) and proposals for more effective intervention.	Qualitative study with an ethnographic approach	People in vulnerable situations	• Supervision and care in supervised consumption sites. • Connection to health services and treatment for drug users.
Perera et al.^33^, 2022 California	To describe the implementation of a hospital-based intervention that provides harm reduction education and supplies to patients through an addiction consultation team at an urban safety-net hospital.	Quantitative study	Adults	• Implementation of harm reduction programs in hospitals. • Provision of medical care to people with substance use disorder.
Morales Loyola^29^, 2020- 2022 Cuernavaca	Promoting social skills through an e-Health educational initiative with a focus on reducing the risks and harms of psychoactive substance use among school-aged adolescents in the State of Mexico.	Nonexperimental study with prepost intervention measurement using a mixedmethods approach	Secondary school adolescents	• Use of digital platforms for education on the risks of substance use. • Prevention of problematic use among adolescents through accessible information. • Education on the risks of substance use through the use of digital platforms.

HIV: Human Immunodeficiency Virus, NHT: Take-home naloxone, SCSs: Supervised consumption sites

**Figure 2:**
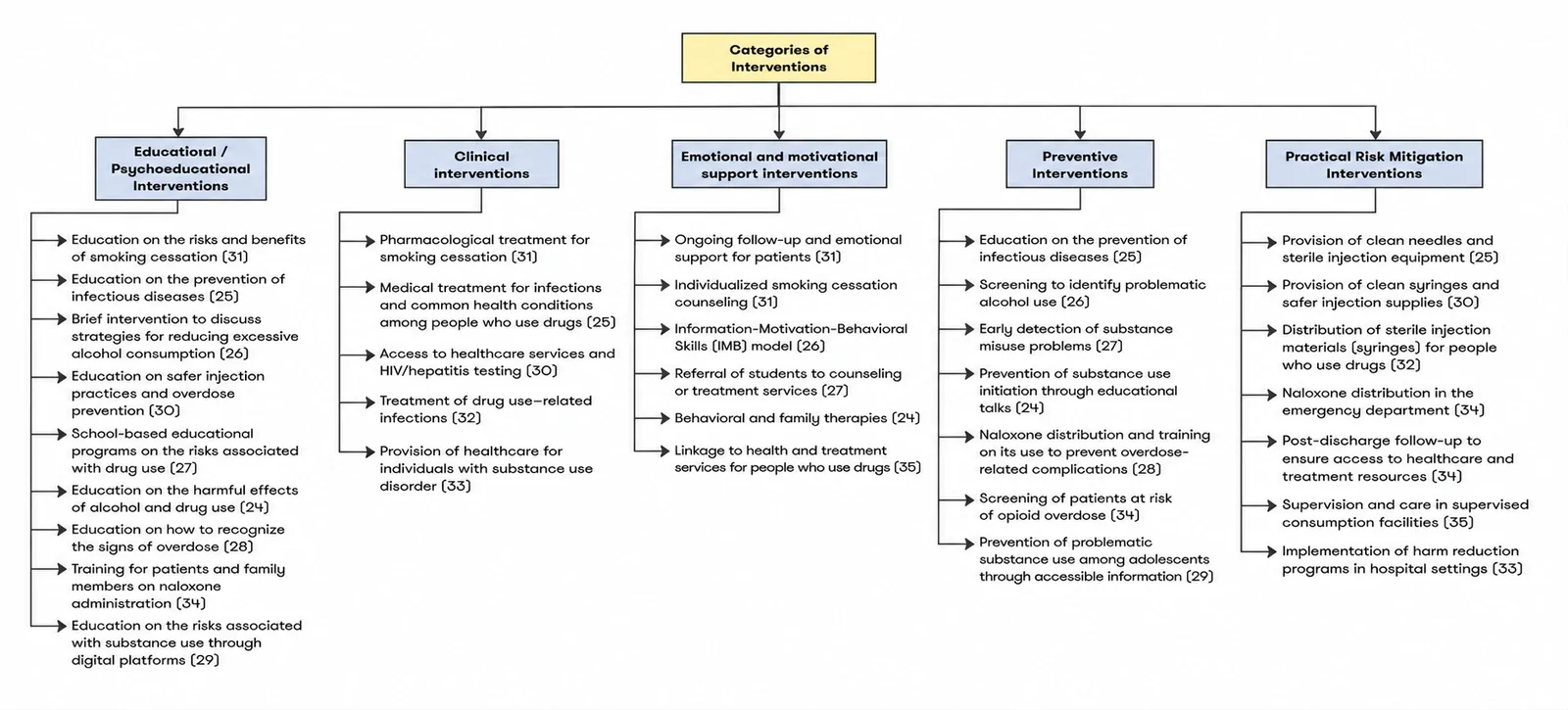
Conceptual map of nursing interventions organized by category

*Categories created based on the authors’ reports and detailed in Supplementary Material^22^*

**Educational or psychoeducational interventions**

The results show that educational interventions led by nursing professionals were the most frequently reported in the included studies. These strategies were primarily aimed at strengthening knowledge about the risks associated with substance use, promoting self-care practices, and harm reduction^24^.

Through educational programs in various settings, they aimed to raise awareness of the risks of tobacco, alcohol, and other drug use using the 5R model (relevance, risks, rewards, roadblocks, and repetition), seeking to increase patients’ motivation to quit^25^,^26^.

In schools, these interventions provided students with a comprehensive understanding of the harmful effects of drugs, leading to a decrease in their use. Additionally, brief interventions were conducted with students at risk of substance use, informing them about the physical, psychological, and social effects of substance use and strengthening their willingness to adopt healthier behaviors^27^.

Other strategies included education on safe injection practices and overdose prevention, training to recognize signs of overdose and the proper use of naloxone, which helped connect users with community treatment and mental health services, strengthening comprehensive care and promoting safer practices^25^,^30^.

**Clinical Interventions**

It was observed that clinical interventions provided by nursing professionals, such as smoking cessation treatment through counseling and pharmacological therapies, proved effective in reducing physical and psychological dependence, increasing motivation to quit using^31^. Likewise, interventions aimed at treating infections and communicable diseases in people who use drugs were described, including sexually transmitted infections, skin lesions, and abscesses associated with injection drug use, through the use of antibiotics, antivirals, and procedures such as incision and drainage, when necessary, along with vaccination programs against Hepatitis B and tetanus.

Furthermore, the integration of medical services—such as testing for infectious diseases, treatments, and management of chronic conditions—significantly improved access to healthcare, promoting improvements in the overall health of the drug-using population and increasing their adherence to treatments^30^. This strategy emerged as a key factor in improving quality of life and reducing barriers to accessing health services^33^. 

**Emotional and Motivational Support Interventions**

These were aimed at strengthening treatment adherence and promoting behavioral changes related to substance use. Early identification of at-risk students facilitated access to support networks within the school setting, making nursing professionals a key entry point for those seeking assistance^27^.

One of the main strategies was the Information-Motivation-Behavioral Skills (IMB) model, which combines information provision with strategies to strengthen motivation and develop behavioral skills that reduce risky behaviors.

Nursing professionals played an essential role in guiding and referring patients to health services, treatment programs, and social resources, promoting a holistic approach that integrates harm reduction and comprehensive medical care^30^, based on the 5A model (ask, advise, assess, assist, and arrange follow-up). They also involved families in the intervention process, strengthening the support network and creating a more favorable environment for recovery^24^.

**Preventive Interventions**

The studies focused on the early detection of substance use and the reduction of risk factors, especially among adolescents and students. Through screening programs and risk identification questionnaires, nursing staff have been able to intervene promptly, preventing problematic patterns from evolving into more serious disorders^26^,^27^. Their work in school settings through educational programs and informational talks, in collaboration with students, parents, and teachers, has been key to promoting healthy lifestyles, strengthening resilience to social pressure, and preventing the onset or continuation of drug use^24^. In addition, the use of digital platforms has been promoted to facilitate informed decision-making and to reinforce general health promotion, including the prevention of communicable diseases and opioid overdoses^29^.

**Practical interventions for risk mitigation**

Harm reduction interventions in the care of people who use drugs, both in hospital and community settings, are essential strategies aimed at minimizing the risks associated with drug use without requiring abstinence. Among the most notable initiatives are sterile syringe exchange programs, which have successfully reduced the transmission of infections such as HIV and hepatitis C, and the distribution of naloxone kits, empowering users and communities to respond to overdoses and effectively save lives^25^,^32^.

Other relevant strategies include supervised consumption sites, where health professionals supervise substance use under safe conditions, and the provision of safe injection kits in hospitals^34^,^35^. These measures not only reduce immediate risks such as overdose and infections but also facilitate access to health services and rehabilitation programs.

## Discussion

The results of this study show that nursing interventions aimed at harm reduction for people who use drugs, developed within the PHC setting, focus on protecting life by strengthening access to health services, as well as on reducing the transmission of HIV and hepatitis B and mortality from overdose. Furthermore, these interventions help protect the dignity of people who use drugs by promoting respect for autonomy and non-stigmatization.

PHC represents individuals’ first point of contact with the healthcare system and enables the creation of ongoing therapeutic relationships with patients^24^,^26^. Barbara Starfield emphasizes that PHC is characterized by providing person-centered, continuous, and coordinated care across different health services—essential elements for adequately addressing the needs of vulnerable groups^36^.

From this perspective, the nursing team plays a fundamental role in implementing strategies such as early detection of substance use, health education, brief counseling, and ongoing follow-up with individuals who use drugs. Martínez Esquivel further emphasizes that nursing professionals who provide substance use counseling must cultivate skills such as active listening, empathy, and assertive communication. These qualities foster a therapeutic relationship based on respect and trust toward the user^16^.

These skills facilitate treatment through an approach that is not stigmatizing but rather understanding, which makes it easier to provide guidance on the risks associated with drug use and to promote safer practices^14^,^15^. In general, these proposals support the essential role of nursing in implementing PHC-based interventions that help reduce the risks and adverse effects associated with drug use, and improve access to health services and the individual’s well-being^15^,^16^.

Among the interventions identified in the literature, health education stands out as one of the most frequently implemented strategies in nursing care for people who use drugs. These actions include providing information on the risks associated with drug use, early identification of signs of overdose, promotion of safer drug use practices, and guidance on timely access to health services. Together, these interventions contribute to increasing individuals’ knowledge, strengthening their capacity for informed decision-making, and reducing the adoption of risky behaviors, which promotes health protection and the prevention of adverse events associated with drug use^26^.

Complementarily, the literature describes specific clinical interventions aimed at preventing overdose deaths, among which the distribution of naloxone accompanied by training in its use stands out. This strategy is primarily implemented in contexts where opioid use constitutes a significant public health problem. Evidence shows that take-home naloxone programs are an effective measure for reducing mortality by enabling an immediate response to potentially fatal events. In these programs, nurses play a fundamental role in educating, training, and monitoring users, which strengthens their ability to act promptly in emergencies related to substance use^25^.

Additionally, prevention interventions focused on strategies for access to supplies for safe use—such as needle exchange programs or the provision of sterile equipment—aim to reduce the transmission of infections like HIV and viral hepatitis, among other complications related to injectable substances. These interventions seek to build trust among users in these settings and strengthen links with other health services^33^,^37^.

Similarly, the reviewed literature highlights the importance of interventions related to accompaniment, counseling, and psychosocial support, which are essential for comprehensively addressing the needs of people who use drugs. These strategies promote adherence to treatment, facilitate access to mental health services, and help reduce the stigma associated with drug use—factors that, collectively, are associated with improvements in quality of life and continuity of health care^26^,^27^.

A comparison of the analyzed studies shows that harm reduction interventions can be implemented in various care settings—including hospitals, community-based settings, or specialized programs—sharing the goal of reducing the negative consequences of drug use without necessarily requiring abstinence. However, the scope and intensity of the interventions vary across studies, making it difficult to clearly establish which ones are most effective or offer the best cost-effectiveness for health systems.

These differences reflect the diversity of approaches within the harm reduction model and highlight the need to adapt interventions to the specific characteristics of the context, available resources, and the particularities of the population served^7^,^38^,^39^.

Among the main limitations of this study is the limited availability of scientific literature on nursing interventions for harm reduction in the primary health care setting, especially in Latin American contexts. This situation restricts the possibility of extrapolating the findings to health systems and sociocultural realities specific to the region. Additionally, it was found that many of the available studies analyze drug use in relation to various comorbidities, which can create methodological difficulties, given that there is not always a direct causal relationship between these conditions and substance use. This interrelationship can hinder the precise identification of nursing interventions specifically aimed at harm reduction, introducing potential biases in the interpretation of the evidence.

The review did not include a systematic assessment of the methodological quality of the selected studies, a factor that should be taken into account when interpreting the results. In addition, a significant proportion of the studies analyzed (approximately 83%) originated in the United States or other developed countries, suggesting the need for cultural, organizational, and structural adaptations for their implementation in Latin American health systems.

Finally, based on the findings identified, it is recommended to strengthen recognition of the role of nursing professionals in the comprehensive care of people who use drugs, promoting greater levels of professional autonomy in clinical decision-making based on scientific evidence. Similarly, it is essential that health policies incorporate strategies aimed at optimizing the use of available resources, prioritizing the prevention of adverse events and the reduction of complications associated with drug use. In this regard, it is necessary to promote new research that delves deeper into the effectiveness of nursing interventions for harm reduction, particularly in Latin American contexts, to generate contextualized evidence that informs the design of more relevant and effective care policies and practices.

## Conclusions

Through a comprehensive analysis of the literature, various interventions carried out by nursing professionals were identified, with education, counseling, emotional support, and clinical treatment being the most prominent. However, specific harm reduction strategies, such as training and the distribution of naloxone for overdose prevention, were less frequently reported in the analyzed studies, highlighting a gap in current research. Nevertheless, these strategies highlight the comprehensive role of nursing, not only in the clinical management of addiction but also in risk prevention through educational and supportive interventions.

It was observed that educational and preventive interventions were the most frequent, particularly among adolescents in school settings, helping to prevent the progression from experimental use to abuse or drug dependence. Likewise, clinical interventions proved to be fundamental in improving treatment adherence and reducing overdose mortality rates. Meanwhile, risk mitigation strategies and emotional support promote users’ well-being and reduce harm associated with drug use. This highlights the role of nursing staff in disciplinary competencies that enable them to lead harm-reduction interventions, ensuring comprehensive care that addresses the biological, psychological, and social dimensions of the individual.

Most of the included studies were conducted in the United States, which limits the transferability of the results to other sociocultural and healthcare contexts. In this regard, it is recommended to promote research in local contexts evaluating harm reduction strategies and nursing-led care models, with the aim of generating contextualized evidence that contributes to strengthening public policies and care programs in the region.

## Supplemental Material

**Supplementary Material 1:** Categories described according to interventions

Category	Intervention	Description
Educational / psychoeducational
	Education on the risks and benefits of quitting smoking.	Intervention known as the 5Rs: relevance; where it is explored whether quitting smoking is personally relevant; risks; where the patient is helped to identify the negative consequences of tobacco use; rewards; where the benefits of quitting tobacco use are identified; roadblocks; where the patient’s barriers or impediments to quitting smoking are identified; and repetition; where it is clarified that this intervention must be repeated in each treatment session; these are most successful when staff are empathetic; promote autonomy; and support the patient’s selfefficacy^31^.
	Education on the prevention of infectious diseases.	Advise all couples on the consistent use of condoms and emphasize adherence to treatment. If arterial or skin lesions such as abscesses; swelling; or severe inflammation are observed; seek medical attention. When injecting drugs; ensure good skin hygiene and handwashing; along with the use of clean injection equipment each time; and recommend reducing the frequency of injections^25^.
	Brief intervention to discuss strategies for reducing excessive alcohol consumption.	After administering the AUDIT questionnaire; provide personalized feedback on the results; offer a health education pamphlet with simple tips; and provide brief counseling on how to reduce excessive alcohol consumption during a 20-minute session over a 3-month period^26^.
	Education on safe injection practices and overdose prevention.	The New Jersey Department of Health contracted a resource center to train nurses on patient care; implementing an orientation program and bimonthly meetings. Nurses received support to identify local resources and establish standing orders for nursing interventions; so they began offering health promotion services; including wound care; counseling on safer injection techniques and overdose prevention; and counseling on safe sex and reproduction^30^.
	Educational programs in schools on the risks of drug use.	School districts should allocate funds to effective educational programs for identifying the risks of drug use; the nurse’s role is to evaluate prevention programs from an evidence-based perspective; make recommendations to administrators; students; families; school staff; and the community; as well as implement these programs to reduce drug use^27^.
	Education on the harmful effects of alcohol and drug use.	Workshops and educational sessions were conducted for adolescents; informing them about the physical; psychological; and social risks associated with alcohol and other drug use. This education also addressed the impact of substance use on brain development; academic performance; and interpersonal relationships^24^.
	Education on how to identify signs of overdose.	Brief intervention (10–30 minutes) with individual participants; which included education on opioid overdose prevention and response; as well as safe use of naloxone. The education consisted of assessing patients’ knowledge of the risk factors for an opioid overdose; how to identify and respond in the event of a suspected overdose; and their knowledge and experience with naloxone. Additionally; concerns and questions regarding the risks and responses to an overdose were addressed; the patient was educated on the use of naloxone to take home; and a consumer information sheet was provided; with a follow-up conducted at 3 months^28^.
	Training for patients and family members on the administration of naloxone.	A 3-minute educational video on naloxone was developed; presenting a realistic scenario that reviewed the signs and symptoms of an opioid overdose and demonstrated how to respond immediately to the situation using a commercially available naloxone nasal spray^34^.
	Education on the risks of substance use through digital platforms.	Through the e-Health approach; digital platforms were used to present information on harm reduction and substance use-related risks; the skills promoted included decision-making; selfefficacy; and resistance to peer pressure^29^.
Clinical interventions
	Pharmacological treatment for smoking cessation.	Nicotine users attempting to quit smoking should receive pharmacotherapy with first-line medications; which include nicotine replacement therapies—such as patches; gum; sprays; and inhalers—that are tapered off as the patient tolerates them without causing withdrawal; and sustained-release bupropion; which should be administered for 8 to 12 weeks with a minimum maintenance period of 6 months^31^.
	Medical treatment for infections and common illnesses among drug users. Vaccination against hepatitis and tetanus.	Management of sexually transmitted infections with antibiotics or antivirals and management of skin lesions with antibiotics and analgesics for pain; or; if necessary; incision and drainage. For tetanus vaccination; 0.5 ml of tetanus toxoid should be administered intramuscularly in the upper arm after the skin is cleaned with an alcohol swab. Notify the patient when the next dose is due; as there are 5 doses (1 month; 6 months; 1 year; and 1 year). For hepatitis B; the dose of Engerix-B and Recombivax HB is 0.5 mL IM up to age 20 or 1 mL IM for adults (≥ 20 years). The dose of Heplisav-B (HepB-CpG) is 0.5 mL IM for adults ≥ 18 years of age; administered in 3 doses. The second dose is administered 1 month after the first dose; followed by a third dose 6 months after the second^25^.
	Access to medical care and HIV/hepatitis testing.	DAs part of the needle exchange program; participants also had access to basic care to reduce barriers to care and receive comprehensive care; therefore; HIV and hepatitis testing were conducted to facilitate early diagnosis and treatment in the event of positive results; ensuring pre- and post-test counseling^30^.
	Treatment of drug-related infections.	Injecting drug users who come to the hospital for infections and require prolonged treatment can be managed with antibiotics; preferably administered via intravenous catheters; as evidence for the efficacy of oral antibiotics is weak; however; caution must be exercised regarding primary infections associated with the intravenous catheter^32^.
	Provision of medical care to people with substance use disorder.	People with substance use disorder should receive medical care at various harm reduction sites; where each patient’s needs are taken into account; providing personalized care that includes support services; diagnostic testing and treatment for hepatitis C and HIV; overdose prevention; naloxone; condoms; and referrals to addiction services^33^.
Emotional and motivational support interventions.
	Ongoing follow-up and emotional support for patients.	Ongoing follow-up and emotional support for patients. The “5A” intervention; in which tobacco users are identified and asked about their use; smokers are then advised to quit; as this benefits their health; the patient’s willingness to attempt to quit is subsequently assessed; the patient is helped to quit through practical advice and social support; and finally; a follow-up program is organized; either in person or by telephone^31^.
	Individualized counseling to quit smoking.	Three components must be taken into account: practical counseling; social support within treatment; and social support outside of treatment. Practical counseling focuses on problemsolving; encouraging recognition of high-risk situations and the development of coping skills; within social support in treatment; the patient is encouraged to discuss the process of quitting smoking; express concern; and demonstrate a willingness to help^31^.
	Information-Motivation- Behavioral Skills (IMB) Model.	In this intervention; in addition to providing information on methods for reducing and preventing harmful use; it is necessary to generate motivation to change behavior and foster behavioral skills that alter risky consumption patterns; which is crucial for harm reduction^26^.
	Connecting students with counseling or treatment services.	In the school setting; the school nurse serves as the link between the student; their family; and the community and health services; particularly when providing drug abuse prevention programs^27^.
	Behavioral and family therapies.	Cognitive-behavioral therapies are conducted with the aim of teaching adolescents coping skills they can use when facing situations and feelings that trigger a return to alcohol or drug use. Additionally; interventions were conducted with family members to identify behavioral and communication patterns that might be related to substance use; including conflict resolution skills and the promotion of a supportive family environment^24^.
	Connection to health services and treatment for drug users.	Within supervised consumption sites; interventions are not only aimed at supervising consumption but also at helping patients access additional health services such as addiction treatment; mental health counseling; and rehabilitation programs; thereby addressing not only the physical but also the emotional and social needs of the user^35^.
Preventive interventions
	Education on the prevention of infectious diseases.	Providing STI counseling and testing. Drop-in centers have been shown to have a positive impact on increasing knowledge among the drug-using population regarding HIV/AIDS prevention; overdose prevention; and other health issues^25^.
	Screening to identify problematic alcohol use.	The Alcohol Use Disorders Identification Test (AUDIT) should be administered; it consists of 10 items assessing levels of alcohol consumption (three items); symptoms of alcohol dependence (three items); and problems associated with alcohol consumption (four items); for a maximum score of 40. Individuals scoring 8 or higher are identified as at-risk drinkers or those with a tendency toward problematic drinking and should therefore receive educational intervention^26^.
	Early detection of substance abuse problems.	Drug abuse screening should begin at school age; therefore; both risk factors and protective factors for drug abuse must be identified; addressed; and influenced within the contexts of the individual; family; community; and society^27^.
	Prevention of substance use initiation through informational talks.	Selective interventions focus on high-risk adolescents; while targeted interventions focus on those who already use substances. Their goal is to reduce substance use and prevent progression. Additionally; early recognition and treatment of mood disorders can prevent substance use; and limiting access to alcohol and other drugs is essential for prevention; since substance use at home is one of the main factors that lead adolescents to want to start using them^24^.
	Distribution of naloxone and training on its use to prevent overdose-related complications.	Providing clients with naloxone to take home. The project used a pre-filled syringe for intramuscular injection (2 ml containing 5 mg/ml). X 0.4 mg dose). This medication was provided free of charge to the user^28^.
	CScreening patients at risk of opioid overdose.	Risk factors for opioid overdose should be identified in individuals presenting to emergency departments. This involves determining whether the patient has any of the following: a history of overdose; a history of substance use disorder; a mental health condition; participation in a detoxification program; use of high doses of opioids; or a prescription for opioid medication^34^.
	Prevention of problematic substance use among adolescents through accessible information.	Central to the approach of e-health interventions is the harm reduction model; whose interventions promote virtual environments as an alternative that brings health services closer to people; with the aim of keeping people alive and motivating them to make positive changes in their lives; preventing experimental use and sporadic use from becoming chronic use; reducing or limiting the harm caused to the health of people who use drugs; and decreasing the undesirable socio-health effects associated with their use^29^.
Practical interventions for risk mitigation
	Provision of clean needles and sterile injection equipments.	Strategies for distributing clean needles and sterile injection equipment were implemented to promote safe injection practices. Additionally; information was provided on the safe use of injectable medications; including not reusing or sharing needles to prevent infections and the spread of communicable diseases; rotating injection sites; etc^25^.
	Provision of clean syringes and safe injection equipment.	Syringe access programs were implemented in five cities in New Jersey. The program consisted of distributing sterile syringes and other injection equipment; such as alcohol swabs; sterile water; and filters; this prevented the sharing of these items; with the aim of promoting safe injection practices and reducing the transmission of infectious diseases^30^.
	Provision of sterile materials (syringes) for people who use drugs.	Needle and syringe programs focus on the distribution of syringes; needle exchange; and the provision of sterile equipment for people who use drugs; it is also important that injection kits be provided during healthcare visits to mitigate the risk of contracting bloodborne infections^32^.
	Distribution of naloxone in the emergency department.	Naloxone should be distributed to adults and adolescents who received care in the emergency department following an opioid overdose; providing a naloxone kit to take home and establishing a standard of care and evidence-based clinical guidelines that include recommendations for the distribution of a commercially prepared 4 mg intranasal naloxone spray; to be prepared for future out-of-hospital overdoses^34^.
	SPost-discharge follow-up to ensure access to care and treatment resources.	Patients who received education through the educational video and were provided with naloxone kits were given access to the video at any time after discharge via a QR code to ensure quick access; it is also important to follow up with individuals who received the kit through medical visits^34^.
	Supervision and care in supervised consumption sites.	Nurses follow established protocols to supervise drug users who visit supervised consumption sites (SCSs) to ensure consumption takes place under hygienic and safe conditions; with the goal of improving injection technique and reducing the incidence of abscesses. Patients learn to space out their drug use; thereby reducing the risk of overdose. Workshops on hygienic venipuncture are conducted; protocols are used to monitor vital signs and administer medications; and cardiopulmonary resuscitation techniques are employed. Additionally; nursing care and health promotion interventions are provided for skin and foot injuries; as well as other conditions related to users’ lifestyles^35^.
	Implementation of harm reduction programs in hospitals.	Harm reduction programs were implemented; which are based on providing supplies and education depending on the drug and route of use: For alcohol; a brochure titled “Rethinking Alcohol Consumption” is provided; which offers advice on safe drinking; such as reducing the number of days consumed; eating before drinking; alternating with water; etc. For tobacco; education is provided; along with toothpicks and gum to reduce the “craving” to smoke. For opioids or stimulants consumed by smoking or inhalation; a Pyrex pipe and rubber mouthpiece are provided; along with education to reduce the need to reuse and share equipment; thereby lowering the risk of infection; for injected opioids or stimulants; in addition to education on overdose prevention; injection kits are provided that contain a needle; tourniquet; alcohol wipes; a sharps container; naloxone; and condoms^33^.
